# *Plasmodium falciparum* Mating Patterns and Mosquito Infectivity of Natural Isolates of Gametocytes

**DOI:** 10.1371/journal.pone.0123777

**Published:** 2015-04-14

**Authors:** Isabelle Morlais, Sandrine E. Nsango, Wilson Toussile, Luc Abate, Zeinab Annan, Majoline T. Tchioffo, Anna Cohuet, Parfait H. Awono-Ambene, Didier Fontenille, François Rousset, Antoine Berry

**Affiliations:** 1 Organisation de Coordination pour la lutte contre les Endémies en Afrique Centrale, Yaoundé, Cameroon; 2 Institut de Recherche pour le Développement, Montpellier, France; 3 Faculté de Médecine et des Sciences Pharmaceutiques, Douala, Cameroon; 4 Université Paris-Sud 11, Villejuif, France; 5 Institut des Sciences de l'Évolution, Montpellier, France; 6 Centre Hospitalier Universitaire de Toulouse, Toulouse, France; 7 Centre de Physiopathologie de Toulouse Purpan, Toulouse, France; Kansas State University, UNITED STATES

## Abstract

*Plasmodium falciparum* infections in malaria endemic areas often harbor multiple clones of parasites. However, the transmission success of the different genotypes within the mosquito vector has remained elusive so far. The genetic diversity of malaria parasites was measured by using microsatellite markers in gametocyte isolates from 125 asymptomatic carriers. For a subset of 49 carriers, the dynamics of co-infecting genotypes was followed until their development within salivary glands. Also, individual oocysts from midguts infected with blood from 9 donors were genotyped to assess mating patterns. Multiplicity of infection (MOI) was high both in gametocyte isolates and sporozoite populations, reaching up to 10 genotypes. Gametocyte isolates with multiple genotypes gave rise to lower infection prevalence and intensity. Fluctuations of genotype number occurred during the development within the mosquito and sub-patent genotypes, not detected in gametocyte isolates, were identified in the vector salivary glands. The inbreeding coefficient *Fis* was positively correlated to the oocyst loads, suggesting that *P*. *falciparum* parasites use different reproductive strategies according to the genotypes present in the gametocyte isolate. The number of parasite clones within an infection affects the transmission success and the mosquito has an important role in maintaining *P*. *falciparum* genetic diversity. Our results emphasize the crucial importance of discriminating between the different genotypes within an infection when studying the *A*. *gambiae* natural resistance to *P*. *falciparum*, and the need to monitor parasite diversity in areas where malaria control interventions are implemented.

## Introduction

*Plasmodium falciparum* transmission relies on its successful development within the mosquito vector where fertilization occurs. However, studies on malaria parasite genetic structure have revealed different mating patterns in multiple epidemiological settings. Large deviations from panmixia were observed in malaria endemic areas and it has been argued that self-fertilization would favor transmission of better adapted strains of parasites. However, the question remains controversial and opened a large debate about the malaria parasite mode of reproduction [1–4]. Deciphering the complexity of *P*. *falciparum* sexuality and its mating pattern would help understanding the disease’s epidemiology and predicting, for instance, the spread of drug resistance.

In malaria endemic areas, multiple genotypes are generally found within a single parasite isolate and the complexity of malaria infections has often impeded genetic studies on *P*. *falciparum* [5,6]. On the vector side, the differential transmissibility of *P*. *falciparum* clones to mosquitoes is poorly known owing to the technical constraints for isolating gametocytes from trophozoites in the blood and for infecting a sufficient number of mosquitoes. However, modeling malaria transmission is now crucial to understand the evolution of parasites and vectors and to predict the long-term impact of global control measures.

In this study, we explored the infectiousness of gametocyte donors and investigated the effect of multiplicities of infection on the mosquito infection success in regards to gametocyte densities. We then assessed the genetic diversity of *P*. *falciparum* during sporogony and characterized the parasites genetic structure from individual oocyst genotypes. The results show how the genetic composition of the gametocyte population impacts on the infection success of the mosquito and describe the important role of the insect vector in maintaining the parasite genetic diversity.

## Materials and Methods

### Ethics statement

Participants were enrolled upon signature of an informed consent by their legal guardian (parent or tutor having legal custody of the child). The experimental and consent procedures were approved by the National Ethics Committee of Cameroon (protocol *#*039/CNE/MP/06).

### Mosquito infections

The survey was conducted over two years, from 2007 to 2008, during the rainy seasons. Procedures for gametocyte carrier detection, blood collection and mosquito infections were performed as previously described [7]. Briefly, *P*. *falciparum* gametocyte carriers were identified among asymptomatic children aged from 5 to 11 in primary schools from the Mfou district, 30 km from Yaoundé (Cameroon). Gametocyte densities read from thick blood smears were expressed as the number of parasites seen against 1000 leukocytes, assuming a standard concentration of 8000 leukocytes per μl. Venous blood was drawn in heparinized Vacutainer tubes in the antecubital fossa. Membrane feedings were set using donor’s blood with replacement of the serum by a non-immune AB serum. Our local laboratory strain of *A*. *coluzzii*, named Ngousso, was used for mosquito feedings. Mosquitoes are reared at the laboratory under standard insectary conditions (27 ± 2°C, 85 ± 5% RH, 12h light/dark).

### Gametocyte isolation

Gametocytes were separated from 1 ml of serum-free blood using MACS columns, as previously described [8], and the gametocyte pellet resuspended in 50 μl of PBS. DNA extractions from purified gametocytes were performed with DNAzol, (Molecular Research Center, Inc., Cincinnati, OH, USA), and resuspended in 50 μl of PBS. A 1 μl volume of gametocyte DNAs was subjected to whole-genome amplification (WGA) using the GenomiPhi V2 DNA Amplification Kit (GE HealthCare, Uppsala, Sweden) according to the manufacturer’s instructions, and resuspended to a 100 μl final volume. All samples were kept frozen at -20°C until further processing.

### Mosquito dissections and *P*. *falciparum* DNA isolation

The blood fed mosquitoes were processed at different stages of *P*. *falciparum* development as follow: 1) to determine the success of infections, a batch of at least 20 mosquitoes was dissected on day 8 post feeding. The midguts were removed and stained in a 0.4% mercurochrome solution and the developed oocysts were counted by light microscopy. The prevalence of infection was defined as the proportion of infected mosquitoes among the total number of dissected mosquitoes and the infection intensity as the number of oocysts per *P*. *falciparum*-positive mosquito. 2) for oocyst genotyping, the midguts were dissected on day 9 after the infected bloodmeal and then placed in 50 μl of absolute ethanol. Individual oocysts were isolated from rehydrated midguts as described in Annan *et al*. [2]. The DNeasy Blood and Tissue Kit (Qiagen, Valencia, CA) was used to purify *P*. *falciparum* DNA from each oocyst according to the manufacturer’s instructions except that elution was carried out with 50 μl of buffer. 3) for sporozoite genotyping, salivary glands were dissected at day 14 post infection and placed in 200 μl of DNAzol. DNA extractions were processed according to the protocol provided by the manufacturer, and the pellet resuspended in 20 μl of sterile water. A *P*. *falciparum* specific PCR (PF1 5’-GGAATGTTATTGCTAACAC-3’ and PF2 5’-AATGAAGAGCTGTGTATC-3’) was carried out on salivary gland DNAs to identify sporozoite positive samples, and DNAs from positive samples from each blood donor were pooled.

### *P. falciparum* genotyping

Genetic polymorphism was assessed for six microsatellite loci according to Anderson *et al*. 2000 [9]. PCR reactions were processed as previously described [2]. The same protocol was used to amplify DNAs from gametocytes, oocysts and salivary glands, using 2 μl of DNA. PCR products were resolved on an ABI Prism 3100 DNA Genetic Analyzer (Applied Biosystems, Foster City, CA) and alleles were read with the GeneMapper software (Applied Biosystems, Foster City, CA). Multiple alleles were scored automatically and inspected visually to correct allele sizing or artifacts. The MOI was determined as the maximum number of alleles at the more polymorphic locus, which provides the minimum number of clones per isolate as the multilocus genotype of each clone cannot be reconstructed from multiple infections.

### Statistical analysis

Statistical analyses were performed using the R statistical software [10]. Isolates with MOI ≥ 4 were pooled, because of the low number of samples for the highest MOIs. The relationship between the distribution of parasites within mosquitoes and the parameters of gametocytes measured from the donor blood, i.e. gametocyte density and MOI, was estimated using a hurdle model [11]. The zero component was used for modeling the infection prevalence using the logit link function, and the count component for the intensity of infection using hurdle negative binomial (HNB). The gametocyte distribution is positively skewed, the mean is bigger than the median, and gametocyte loads were log-transformed. Maximum likelihood estimates were obtained using the glmmADMB package in R [12] and optimal models were selected using the Akaike Information Criterion (AIC). Goodness of fit was tested using a chi-squared test comparing the observed and predicted oocysts counts [13].

### Genetic structure of *P*. *falciparum*

Genetic analysis was performed on individual genotypes of oocysts isolated from mosquitoes fed on blood from nine different gametocyte carrier donors. We considered three levels: 1) oocysts within a mosquito midgut, the subpopulation level; 2) oocysts from a single blood donor, the population level; and 3) all oocysts, the metapopulation level. The observed (*H*_O_) and expected (*H*e) heterozygosities under random mating were estimated with the FSTAT 9.2.4 program [14] using the unbiased estimator of Nei [15], which corrects for small samples. We measured the frequencies of selfing in mosquitoes fed on individual blood donors. Selfing was defined as mating between two genetically identical gametes; i.e., we assumed that oocyst genotypes that are homozygous at all six loci resulted from self-fertilization.

Wright’s *F* statistics were calculated according to Weir and Cockerham’s estimators [16] using the GenePop 4.0 and FSTAT 9.2.4 packages [14,17]. *F*is gives a measure of the correlation of the allelic type within oocysts relative to gene copies from different oocysts from the same mosquito. Deviations from Hardy-Weinberg expectations (*F*is = 0), were tested by randomizing alleles between oocysts from a single midgut. *F*st measures the correlation of the allelic type among different oocysts from the same mosquito relative to gene copies in oocysts from different mosquitoes fed on the same donor blood. The genetic differentiation between oocyst subpopulations (midgut level) within each oocyst population (donor level; *F*st > 0) was tested by randomizing oocyst genotypes for each donor. The linkage disequilibrium between pairs of loci was tested by exact log-likelihood *G* tests using 10,000 permutations and Bonferroni correction.

## Results

### Parameters from the *P*. *falciparum* gametocyte population that affect parasite transmission by the mosquito vector

We performed 139 experimental feedings to infect female mosquitoes of the *A*. *gambiae* Ngousso strain with gametocyte-containing blood from naturally infected human volunteers. The mean gametocyte density among blood donors was 122 ± 119 per μl (range: 11–2,304). We dissected 6,227 mosquito midguts at day 7 post infection over the 139 feedings. For 14 feedings (10%) no oocyst was detected, and these infections were not further analyzed. Among the 125 successful infectious blood meals, the infection prevalence varied from 10.8 to 100.0% (mean: 68.7 ± 9.4) and the mosquito infection intensity per gametocyte donor from 1.13 to 226.84 oocysts per mosquito (median: 7.28, IQR 4.02–17.61; S1 Table). The multiplicity of infection (MOI) in the gametocyte samples ranged from 1 to 10 clones (mean: 3.12 ± 0.84) and 17 gametocyte donors had monoclonal infections (13.6%).

**Fig 1:**
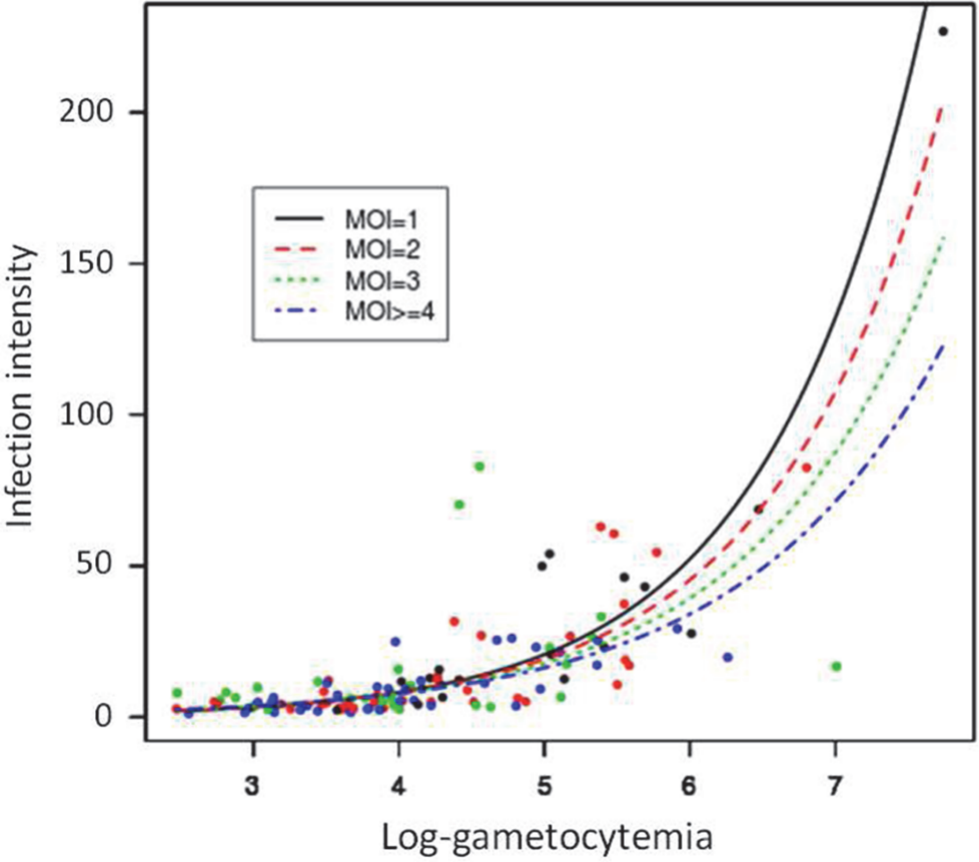
A, fitted intensity of infection curve versus log-transformed of gametocytemia for the different MOIs in the HNB2 model. The analysis was performed from 125 feedings.

**Fig 2:**
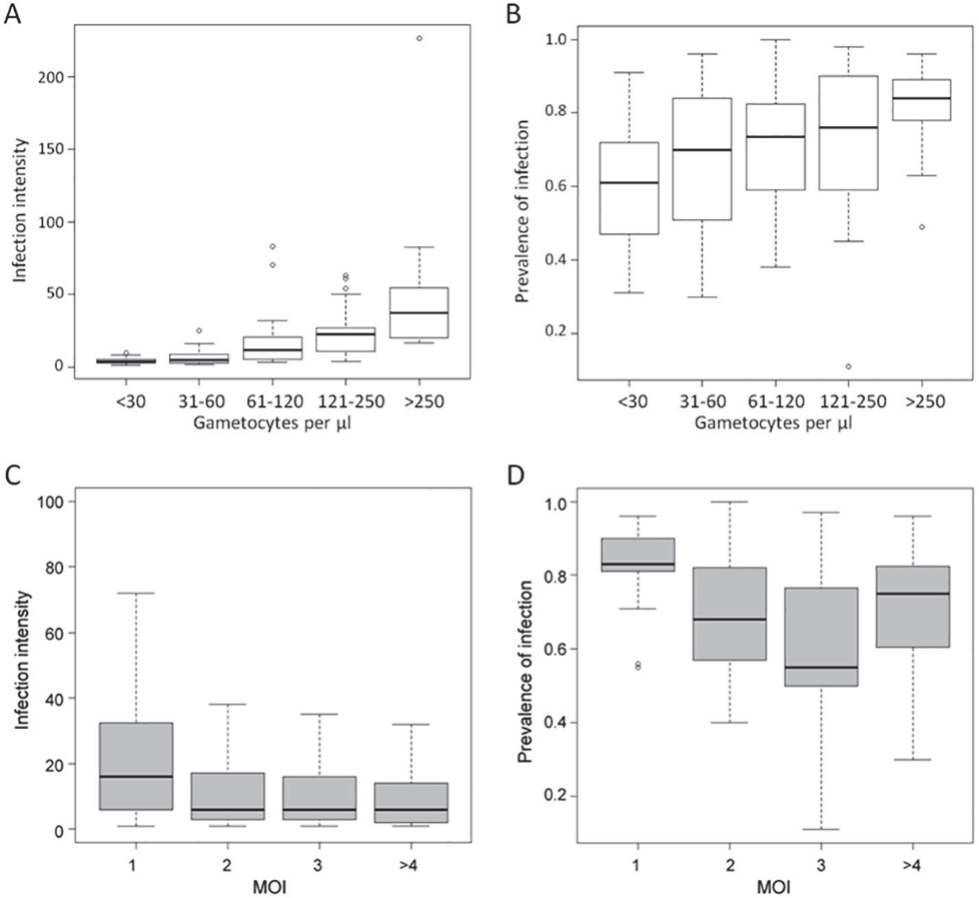
A and B, relation between gametocytes density in the parasite isolate and infection prevalence and infection intensity. Gametocyte densities were grouped into five classes, as indicated in the x axis. Infection prevalence is given as the mean proportion of infected mosquitoes, in percentage, and infection intensity as the median of oocyst counts per *P*. *falciparum*-positive gut. The boxplots were created from 125 feedings. The number of infected mosquitoes and the oocyst loads increase with the gametocyte density in the blood donor (hurdle model, *P* < 2e-16 for both response variables). **C and D, relation between multiplicity of infection in the parasite isolate and infection intensity and infection prevalence.** Multiplicity of infection (MOI) is defined as the maximum number of alleles at the most polymorphic locus. Volunteers harboring MOI >4 were grouped. Infection prevalence is given as the mean proportion of infected mosquitoes, in percentage, and infection intensity represents the median of oocyst counts per *P*. *falciparum*-positive gut for the given MOI class. The boxplots were created from 125 feedings.

The estimates of the parameters of the hurdle negative binomial (HNB) model are shown in Table 1 for positive count and zero components. Both gametocyte density and MOI significantly affected the oocyst number. The fit of the intensity of infection with gametocyte densities for the different MOIs is plotted in Fig 1. In the HBN model, the correlations of oocyst counts with gametocyte density and MOI were positive (*P* < 2e-16 and *P* = 0.0151, respectively; Table 1) and the model showed a significant and negative combined effect of the explanatory variables on the oocyst counts (*P* = 0.0029, Table 1). The number of oocysts in the mosquito midgut increased with the number of ingested gametocytes while increased oocyst loads were observed when MOI = 1 (Fig 2A and 2C). For infection prevalence, the model indicated that both gametocyte density and MOI were highly significant (*P* < 2e-16 and *P* = 4.2e-04, respectively), and the interaction was not significant (Table 1). The relation between gametocyte densities and the number of infected mosquitoes was positive, indicating that the higher gametocytemia the blood donor had, the higher the prevalence of infection (Fig 2B). In contrast, the effect of MOI was negative, which meant that infection prevalence was lower in multiclonal isolates (Fig 2D).

**Table 1:** Maximum likelihood estimates of the parameters in the Hurdle Negative Binomial model.

**For infection intensity (hurdle model)**					
**Parameter**	**Estimate**	**Std. Error**	**z value**	**Pr(>|z|)**	
Intercept	-1.8322	0.2750	-6.66	2.7e-11	***
LogGto	0.9887	0.0577	17.15	< 2e-16	***
MOI	0.2358	0.0970	2.43	0.0151	*
interaction	-0.0631	0.0212	-2.97	0.0029	**
Dispersion	0.66957	0.027334			
**For infection prevalence (logit link function)**					
LogGto	0.2440	0.0192	12.69	< 2e-16	***
MOI	-0.1574	0.0447	-3.53	4.2e-04	***
interaction	0.0234	0.0133	1.76	0.078	ns

**LogGto**, logged-transformed gametocyte density; **MOI**, multipicity of infection; **Interaction** for interaction between LogGto and MOI. The dispersion parameter that is accommodated in the HNB model reflects over-dispersion of parasites. Z value is a Student statistics. Significant codes

'***' <0.001

'**' <0.01

'*' <0.05; 'ns' ≥0.05.

### *P*. *falciparum* genetic diversity throughout the development within the mosquito vector

*P*. *falciparum*-positive salivary glands were pooled for 49 feedings, which led to 49 bulks from 547 sporozoite-positive salivary glands. The proportion of monoclonal infections was similar for the *P*. *falciparum* gametocyte isolates and the sporozoite populations, 14.3 and 8.2%, respectively (odds ratio: 1.86, 95% CI: 0.43–9.32, *P* = 0.524). The mean multiplicity of infection at the gametocyte and sporozoite stages varied from 3.57 ± 0.99 (range: 1–10) to 3.94 ± 0.88 (range: 1–8), respectively, and the difference was not significant (Wilcoxon matched pairs ranks test, *V* = -164, *P* = 0.155).

**Fig 3:**
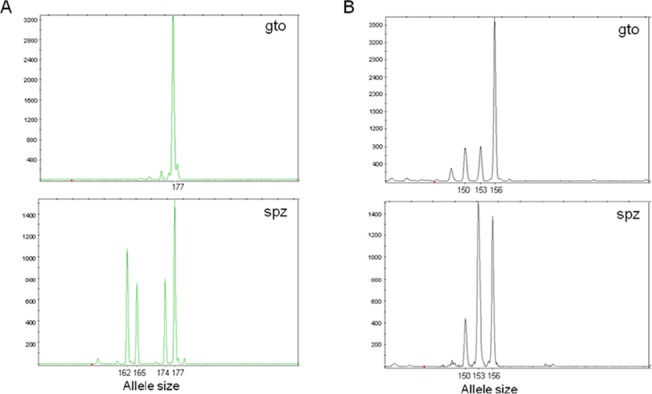
Comparison of *P*. *falciparum* allelic diversity in gametocyte (gto) and sporozoite (spz) populations from the same isolates. **A**, donor CM066 at locus TA109; **B**, donor CM085 at locus PfPK2.The x-axis indicates the allele size at the given microsatellite locus; the y-axis represents arbitrary fluorescent units. Electrophoregrams show the parasite dynamics, from blood (gto) at the time of mosquito feeding to salivary glands (spz). Alleles not detected in the sexual stages are found in the sporozoites (A). The relative abundance of the different genotypes, determined by the height of the fluorescent units, varies between the gto and the spz populations (B), which reflects intense competitive interactions between parasite clones.

We observed different patterns of microsatellite polymorphism between the gametocyte and sporozoite populations for the same parasite donor. The fluorescence peak, which reflects the relative abundance of the alleles, varied between the income and outcome parasite stages, gametocytes and sporozoites, respectively, and most blood donors encountered changes in their allelic composition between the gametocyte and sporozoite populations (Fig 3). Indeed, for 87.8% (43 of 49) of the infections, we observed in sporozoite populations the presence of alleles not detected in the gametocyte population. Genotypes not detected at early stages of sporogony can reach detection level at a later stage, and this can reflect either an inaccurate assessment of the genotypes in the gametocyte samples or gametocyte genotypes present below the PCR threshold.

### Correlation of *P*. *falciparum* mating patterns with parasite transmission

A total of 623 oocysts from 108 mosquito midguts infected on 9 blood donors were successfully analyzed with 6 microsatellite markers. As for the haploid forms of *P*. *falciparum*, all 6 loci were highly polymorphic, with an average of 11.3 (±2.1) alleles per locus. The mean allelic richness of oocyst populations within blood donors was 3.6 ± 1.9. The total observed and expected gene diversities over all 6 loci were *H*o = 0.291 (±0.039) and *H*e = 0.404 (±0.045), respectively. However, significant differences between *H*o and *H*e were not observed for all gametocyte carriers, indicating that inbreeding levels vary from one blood donor to another.

**Fig 4:**
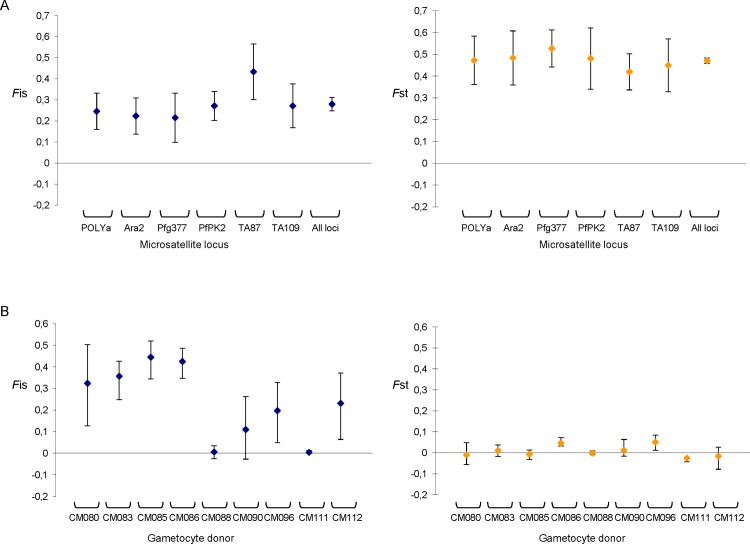
Inbreeding statistics for *P*. *falciparum* oocysts recovered from *A*. *gambiae* midguts per microsatellite locus (A) and per blood donor (B). *F*is gives a measure of the correlation of allelic types within oocysts relative to gene copies from different oocysts from the same mosquito. *F*st measures the correlation of the allelic type among different oocysts from the same mosquito relative to gene copies in oocysts from different mosquitoes fed on the same donor blood. The bars indicate the 95% CI for each value.

*F* statistics were computed to explore the genetic structure of *P*. *falciparum* oocysts within and among mosquitoes fed on blood from the nine distinct gametocyte carrier volunteers. At the meta population level (all mosquitoes), *P*. *falciparum* diploid stages exhibit a non-random distribution within the mosquito vector (*F*st = 0.471, 95% CI: 0.452–0.487, *P* < 10–4) and a significant departure from panmixia (*F*is = 0.280, 95% CI: 0.234–0.339, *P* < 10–4). The genetic differentiation among oocyst sub-populations (*F*st) and the inbreeding coefficient (*F*is) were computed at each microsatellite locus for the whole set of mosquito midguts (Fig 4A); the values were similar to those previously described for populations of *P*. *falciparum* from different areas [2,4]. The linkage disequilibrium was significant for all 15 pairs of loci, with *P* < 10–3 for each pair after Bonferroni correction. The *F*st values per pair of samples (donors) varied from 0.162 to 0.933 and all pairwise values were highly significant.

At the donor level, a significant genetic differentiation between mosquitoes fed on the same donor was observed for only two gametocyte carriers: CM086 and CM096 (*F*st = 0.047, 95% CI: 0.021–0.062; and *F*st = 0.051, 95% CI: 0.017–0.090, respectively, Table 2); genotypes were randomly distributed among mosquitoes for all the remaining blood donors. Departure from panmixia due to non-random mating of gametes within mosquito midguts was then estimated by the *F*is measures for each of the 9 blood donors. The *F*is values ranged from 0.004 to 0.454 over the nine feedings, and *F*is was not significant for only 3 gametocyte carriers (CM088, CM111, and CM090) (Fig 4B and Table 2), which confirmed variations in inbreeding levels according to the blood donor.

**Table 2:** *F* statistics for each of the nine experimental feedings.

Isolate	density	MOI	IP (%)	median [range]	N(guts)	N(ooc)	selfing	*H*o	*H*e	*F*st (95% CI)	*P*	*F*is (95% CI)	*P*
CM080	33.33	7	93.3	11.0 [1–30]	7	28	0.46	0.312	0.477	-0.01 (-0.068. 0.036)	ns	0.324 (0.145. 0.521)	***
CM083	370.37	6	88.9	24.0 [1–103]	10	74	0.17	0.377	0.627	0.012 (-0.014. 0.041)	ns	0.357 (0.287. 0.466)	***
CM085	107.14	4	83.3	23.0 [2–76]	7	88	0.53	0.26	0.429	-0.006 (-0.026. 0.020)	ns	0.445 (0.370. 0.546)	***
CM086	53.33	5	95.7	22.0 [2–61]	10	90	0.38	0.267	0.51	0.047 (0.021. 0.062)	***	0.425 (0.363. 0.503)	***
CM088	43.96	6	79.0	2.0 [1–16]	21	91	0.18	0.64	0.641	-0.001 (-0.012. 0.007)	ns	0.006 (-0.023. 0.037)	ns
CM090	68.18	2	81.3	3.0 [1–16]	15	47	0.89	0.042	0.051	0.012 (-0.040. 0.040)	ns	0.11 (-0.043. 0.247)	ns
CM096	98.48	6	78.6	9.0 [1–36]	16	93	0.39	0.233	0.286	0.051 (0.017. 0.090)	**	0.197 (0.066. 0.345)	***
CM111	73.73	1	83.3	4.5 [1–18]	9	82	0.95	0.025	0.025	-0.025 (-0.028. -0.007)	ns	0.004 (-0.005. 0.006)	ns
CM112	47.06	7	85.4	9.0 [1–25]	13	30	0.18	0.435	0.579	-0.016 (-0.059. 0.046)	ns	0.231 (0.090. 0.398)	***

**Fig 5:**
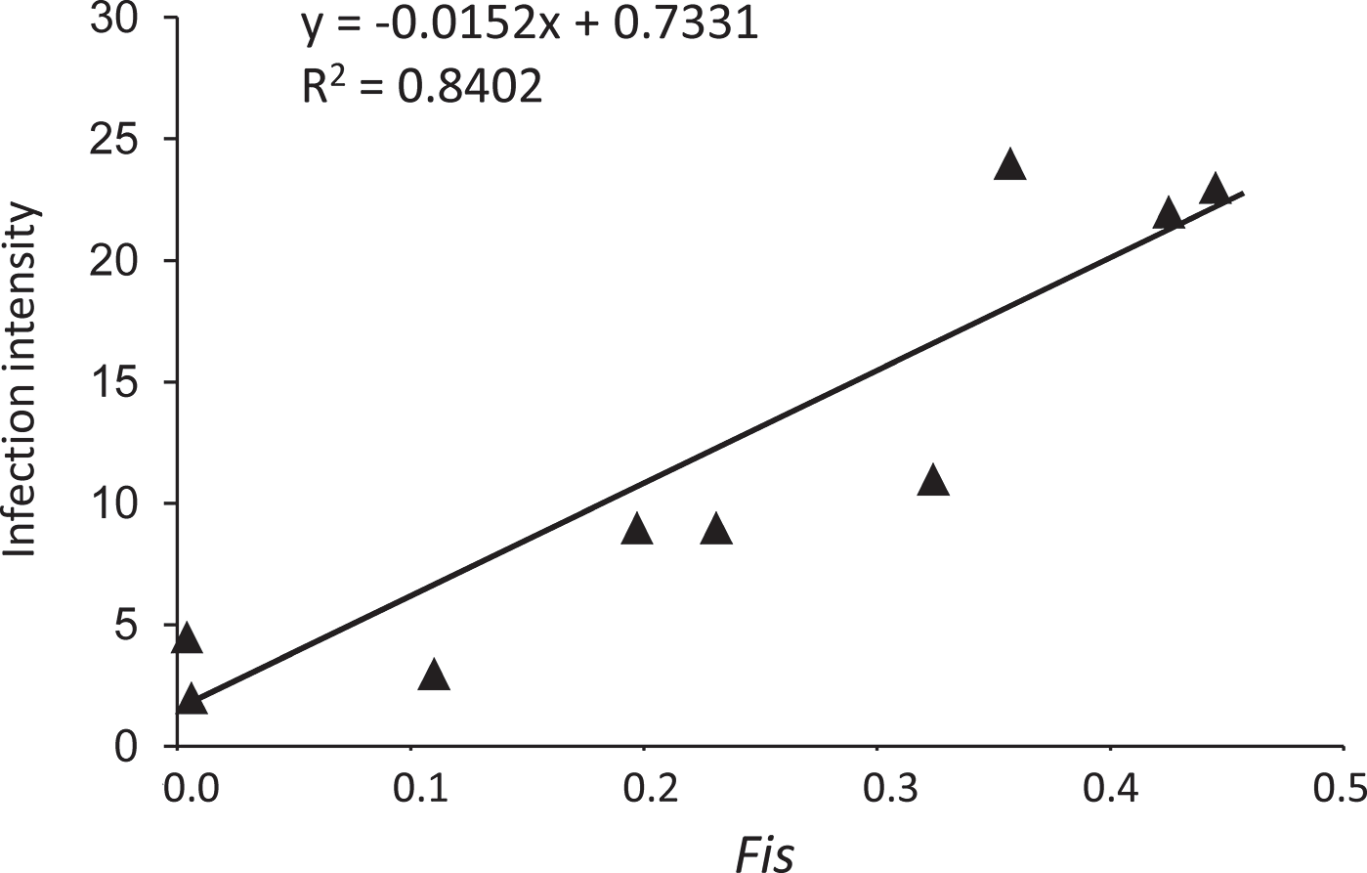
Relationship between the median of the oocyst burden in *P*. *falciparum*-positive mosquitoes and *F*is. Each dot represents a blood donor. The oocyst loads are positively correlated to the *F*is values (*P* = 5.1e-4).

We computed a linear regression model to investigate correlations of *Plasmodium* mating patterns with parameters of infection success. The *Fis* was positively correlated to the medians of oocyst loads (R^2^ = 0.840, *P* = 5.1e-4; Fig 5), indicating that infection intensity is higher when parasites do not mate randomly within the mosquito. We explored whether mating between clone relatives, as seen here by selfing (measured by identical genotypes at all 6 loci in the oocyst population), results from the parasite complexity, and indeed a linear regression model showed that selfing negatively correlates with MOI (R^2^ = 0.786, *P* = 9.0e-4; S1 Fig).

## Discussion

In this study, we have modeled the mosquito infection (infection intensity and infection prevalence) according to gametocyte variables (gametocyte density and MOI) to provide insights into the transmission of *P*. *falciparum* genotypes in the field. Our results indicate that 1) the number of co-infecting clones in the gametocyte isolate impacts on the mosquito infection success, both in terms of prevalence and intensity of infection; and 2) sporogony within the mosquito vector has an important role in shaping the population structure of *P*. *falciparum*.

We performed membrane feedings on blood from asymptomatic gametocyte carriers identified in an area where malaria is hyper-endemic and the entomological inoculation rate exceeds 100 infected bites/person/year [18,19]. A large variability in the mosquito infection success was observed from one feeding to another. We then examined how the parameters from the gametocyte isolates influence the mosquito infection, by scoring gametocyte densities microscopically and genotyping gametocyte isolates using microsatellite markers. Our results show that not only gametocyte density but also MOI have an effect on infection outcomes. The gametocyte density strongly correlates with infection prevalence and intensity: mosquitoes fed on carriers with the highest gametocyte counts were more infected and carried more oocysts. The relationship between gametocyte density and transmission success has already been reported [20,21]; however, the influence of the multiplicity of infection of *P*. *falciparum* gametocyte isolates on mosquito infection parameters has been poorly documented. We found in our studied area that the number of clones within natural gametocyte isolates negatively influenced the infection prevalence and the oocyst loads were higher in monoclonal infections, indicating that coinfections impact on the transmission success.

The transmissibility of multiple clones to mosquitoes, even when present at low density, has already been reported [20,22–24]. The current literature predicts that within-host competition between conspecific parasites results in a competitive advantage for virulent clones [25–27]. However, virulence in these studies refers to a strategy to optimize host exploitation, which is not the only parasite adaptation underlying competition in mixed infections [28,29]. Indeed, parasite plasticity and interactions with the host immunity also play an important role in determining transmission success from coinfecting clones [26,30]. Accordingly, on the vector side, we previously reported that the immune responses mounted by the mosquito differed between monoclonal and multiclonal infections [31]. In the present study and in a previous one on a smaller set of feedings [31], we showed that mixed infections lead to a lower parasite burden. By contrast, a recent study reported that coinfection of two cultured strains of parasites did not affect the mosquito infection [32]. These findings may indicate that the reduced infection level we observed in mixed infections results from former parasite-parasite interactions within the human host and not from vector-parasite interactions. This would explain, in part, discrepancies between studies using wild isolates and those using laboratory strains of *P*. *falciparum* [33,34]. Alternatively, the genetic diversity of the parasite may be an important factor to infect different species of sympatric mosquitoes and further studies will have to determine whether different vector species better transmit specific *P*. *falciparum* genotypes [2,35]. b Increasing evidence also suggests that the mosquito genotype by parasite genotype and genotype by environment interactions are a strong determinant of vector competence [36,37]. Our results highlight the great complexity of parasite-parasite and host-parasite interactions *in natura* and emphasize that both fundamental and field studies will be necessary to measure the importance of parasite and vector, and their biological and genetic features, in driving parasite transmission.

We found here that the overall genetic diversity of sporozoite populations was congruent with that of gametocyte isolates. The maintenance of genetic diversity throughout the sporogony is consistent with previous studies that reported similar patterns of genetic diversity in gametocytes and infected mosquitoes, as seen in pooled oocysts within the midgut [23]. However, we observed in oocyst and sporozoite populations alleles that were not detected in the corresponding gametocyte samples, reflecting the imperfect detectability of minority clones [38]. In our study, the presence of new microsatellite alleles in oocysts and salivary glands of mosquitoes that fed on apparently mono-infected gametocyte carriers indicates that clones were present, but not detected, in the gametocyte population and, more importantly, that they were able to infect mosquitoes. Several studies have reported failure to detect minority clones in blood samples because the sensitivity of molecular methods is not optimal or the numerically dominant clones may obscure the less abundant ones [22–24,38,39]. This limitation probably underestimates the MOI in the gametocyte population and the correlation we found between the gametocyte complexity and the mosquito infection parameters may slightly differ *in natura*. Nonetheless, our results thus confirm that *P*. *falciparum* gametocyte genotypes with sub-patent densities contribute to disease transmission [5,40].

The mating patterns of *P*. *falciparum* determine the parasite population genetic structure, with important epidemiological consequences. Indeed, the dynamics of parasite transmission and the underlying spread of drug-resistant genotypes are directly dependent on the organism’s mode of reproduction [41]. Non-random mating of gametes within the mosquito bloodmeal (significant *F*is) was detected for a majority of feedings (6 of 9) despite random distribution of gametocyte genotypes among mosquitoes (non-significant *F*st). Interestingly, oocyst burden was *F*is-dependent, suggesting that the mating pattern of *P*. *falciparum* influences genotype transmission. Herein, we propose that *P*. *falciparum* is capable of modulating inbreeding and outcrossing levels according to the genetic content of the gametocyte pool or to other environmental cues. This is in agreement with the recent hypothesis that malaria parasites use kin discrimination to gauge the genetic diversity within the infection and adjust their sex-allocation in response to the presence of coinfecting genotypes [42]. The increased oocyst burden found in infections deviating from panmixia may reflect that mating between clone-mates gives rise to progenies with higher fitness, which is consistent with the higher infection intensity observed in monoclonal infections. However, from our oocyst genotyping data, it remains unclear how the genetic content of gametocyte isolates influences the outcrossing level. Further investigations aiming at measuring both the sex ratios and the densities of the different coinfecting genotypes are needed to better understand the transmission strategies in multiclonal infections.

Finally, these results are of importance for the understanding of vector-parasite interactions in the field, as we showed that the genetic composition of the gametocyte population affects the outcome of the infection in the mosquito vector. The current deployment of malaria control interventions should reduce the diversity of circulating *P*. *falciparum* parasite strains, and lead to an increase of monoclonal infections that have better infectiousness for the mosquito vector. Our findings are then of great significance since they suggest an important epidemiological consequence of control interventions. This study shows the importance of monitoring and characterizing malaria infections to understand the changes in malaria epidemiology within the context of malaria control interventions, and to circumvent their unintended effect on vector transmission.

## Supporting Information

S1 Fig Relationship between MOI and selfing. Each dot represents a blood donor. MOI represents the estimated number of clones per gametocyte carrier, Selfing is defined as mating between two genetically identical gametes.
(TIF)

S1 Table Characteristics of the gametocyte donors and parameters of transmission success in the Ngousso strain of *A*. *gambiae*. (DOC)
