# Supplementary figures and images for: Plasmodium falciparum Mating Patterns and Mosquito Infectivity of Natural Isolates of Gametocytes
Source: PLoS One. 2015 Apr 14;10(4):e0123777. doi: 10.1371/journal.pone.0123777 (PMC4397039; doi:10.1371/journal.pone.0123777)

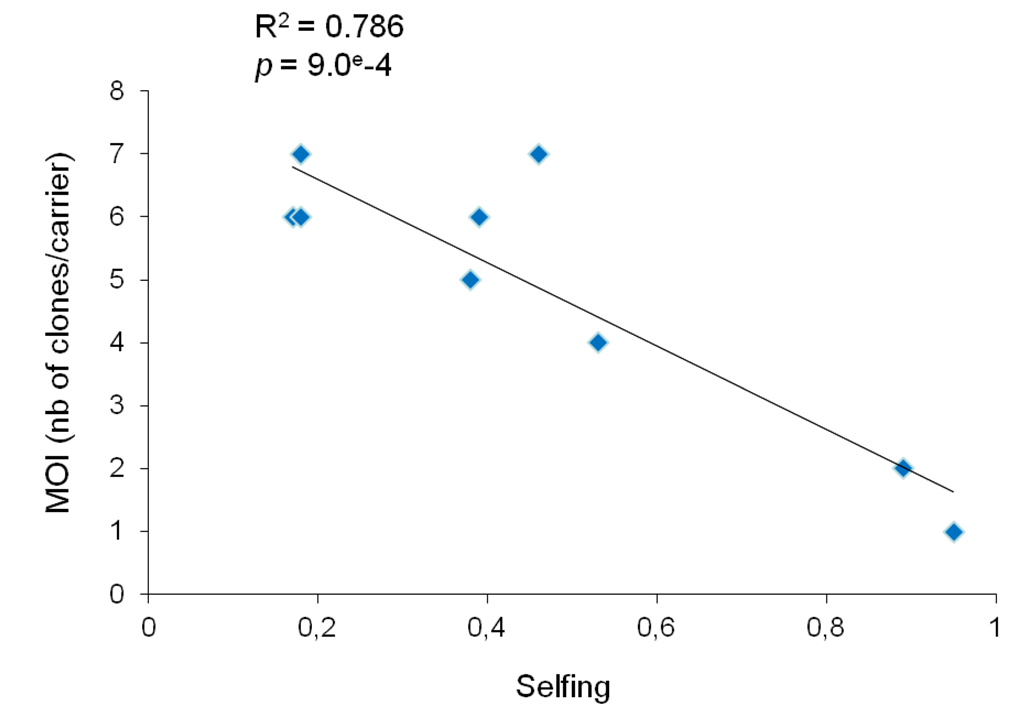

Supplement: S1 Fig — Each dot represents a blood donor. MOI represents the estimated number of clones per gametocyte carrier, Selfing is defined as mating between two genetically identical gametes. (TIF) [file pone.0123777.s001.tif]
