# Supplementary material for: Plasmodium falciparum Mating Patterns and Mosquito Infectivity of Natural Isolates of Gametocytes
Source: PLoS One. 2015 Apr 14;10(4):e0123777. doi: 10.1371/journal.pone.0123777 (PMC4397039; doi:10.1371/journal.pone.0123777)
Supplement: S1 Table — (DOC) [file pone.0123777.s002.doc]

**Table S1. Characteristics of the gametocyte donors and parameters of transmission success in the Ngousso strain of *A. gambiae.***

|  | **Gametocytes**  **(0 dpi)** | | **Midgut infection parameters**  **(8 dpi)** | | | | **Sporozoites**  **(14 dpi)** | |
| --- | --- | --- | --- | --- | --- | --- | --- | --- |
| **donor** | **density** | **MOI** | **n/N** | **IP (%)** | **II (95% CI)** | **median [range]** | **N** | **MOI** |
| CM007 | 900.6 | 2 | 22/45 | 48.9 | 82.50 (48.27, 116.7) | 61.5 [1-250] | 9 | 2 |
| CM013 | 31.4 | 3 | 20/39 | 51.3 | 11.70 (6.11, 17.29) | 6.5 [1-41] | 7 | 3 |
| CM014 | 16.0 | 3 | 16/47 | 34.0 | 5.56 (3.08, 8.05) | 5.0 [1-16] | 8 | 3 |
| CM017 | 154.1 | 3 | 4/37 | 10.8 | 23.00 (0.38, 45.61) | 17.5 [13-44] | 5 | 3 |
| CM024 | 22.9 | 4 | 22/32 | 68.8 | 4.95 (3.51, 6.40) | 4.5 [1-12] | 9 | 4 |
| CM025 | 153.8 | 1 | 21/26 | 81.0 | 54.00 (36.53, 71.47) | 56.0 [1-132] | 11 | 3 |
| CM026 | 2303.7 | 1 | 31/38 | 82.0 | 226.84 (163.0, 290.66) | 200.0 [3-600] | 11 | 1 |
| CM035 | 35.8 | 5 | 40/47 | 85.1 | 7.28 (5.78, 8.77) | 6.5 [1-19] | 9 | 5 |
| CM047 | 245.3 | 2 | 24/41 | 58.5 | 10.71 (0.38, 45.61) | 9.0 [1-32] | 11 | 5 |
| CM048 | 49.2 | 2 | 24/32 | 75.0 | 3.04 (2.07, 4.01) | 2.0 [1-8] | 12 | 4 |
| CM049 | 523.6 | 5 | 39/48 | 80.9 | 19.79 (14.09, 25.49) | 16.0 [1-70] | 8 | 3 |
| CM050 | 20.7 | 4 | 52/74 | 70.3 | 4.92 (3.94, 5.90) | 4.0 [1-14] | 16 | 6 |
| CM052 | 54.9 | 3 | 23/43 | 53.5 | 2.52 (1.83, 3.21) | 2.0 [1-6] | 9 | 3 |
| CM053 | 64.0 | 5 | 20/38 | 52.6 | 7.75 (3.45, 12.05) | 4.0 [1-37] | 11 | 4 |
| CM056 | 173.3 | 3 | 53/65 | 81.5 | 17.60 (13.41, 21.79) | 13.0 [2-73] | 13 | 4 |
| CM060 | 46.2 | 2 | 42/50 | 84.0 | 4.81 (3.59, 6.02) | 4.0 [1-15] | 12 | 5 |
| CM063 | 57.1 | 5 | 49/54 | 90.7 | 9.12 (7.21, 11.03) | 10.0 [1-34] | 9 | 4 |
| CM064 | 647.3 | 1 | 18/23 | 78.3 | 68.67 (36.97, 100.36) | 58.5 [2-227] | 12 | 3 |
| CM066 | 296.3 | 1 | 43/51 | 84.3 | 43.12 (32.78, 53.46) | 34.0 [2-165] | 13 | 4 |
| CM067 | 165.3 | 6 | 31/33 | 93.9 | 21.45 (15.65, 27.25) | 17.0 [1-71] | 12 | 7 |
| CM068 | 55.6 | 2 | 23/28 | 82.1 | 11.74 (8.07, 15.40) | 9.0 [1-35] | 16 | 5 |
| CM069 | 102.6 | 3 | 20/38 | 52.6 | 3.35 (2.16, 4.54) | 2.0 [1-10] | 14 | 8 |
| CM070 | 47.6 | 3 | 34/46 | 73.9 | 4.21 (3.11, 5.30) | 3.0 [1-13] | 16 | 8 |
| CM071 | 62.3 | 1 | 35/49 | 71.4 | 4.14 (3.18, 5.10) | 3.0 [1-10] | 8 | 1 |
| CM072 | 33.3 | 2 | 17/35 | 48.6 | 4.82 (3.46, 6.19) | 5.0 [1-9] | 11 | 3 |
| CM076 | 266.2 | 3 | 42/50 | 84.0 | 17.10 (13.60, 20.59) | 14.5 [1-46] | 16 | 4 |
| CM078 | 23.1 | 4 | 45/50 | 90.0 | 6.51 (3.06, 9.96) | 4.0 [1-79] | 10 | 4 |
| CM079 | 32.2 | 2 | 21/23 | 91.3 | 5.62 (4.15, 7.09) | 5.0 [1-13] | 9 | 3 |
| **CM080** | 33.3 | 7 | 42/45 | 93.3 | 11.21 (9.12, 13.31) | 11.0 [1-30] | 8 | 3 |
| CM081 | 19.5 | 6 | 24/40 | 60.0 | 2.96 (2.13, 3.79) | 2.5 [1-9] | 14 | 7 |
| **CM083** | 370.4 | 6 | 48/54 | 88.9 | 29.27 (23.49, 35.05) | 24.0 [1-103] | 11 | 6 |
| **CM085** | 107.1 | 4 | 25/30 | 83.3 | 25.48 (18.63, 32.33) | 23.0 [2-76] | 12 | 4 |
| **CM086** | 53.3 | 5 | 66/69 | 95.7 | 24.94 (21.25, 28.63) | 22.0 [2-61] | 12 | 5 |
| **CM088** | 44.0 | 6 | 30/38 | 79.0 | 3.75 (2.39, 5.07) | 2.0 [1-16] | 12 | 6 |
| CM089 | 29.1 | 4 | 25/34 | 73.5 | 3.76 (2.25, 5.27) | 3.0 [1-15] | 9 | 3 |
| **CM090** | 68.2 | 2 | 52/64 | 81.3 | 3.88 (3.04, 4.73) | 3.0 [1-16] | 8 | 2 |
| CM093 | 257.1 | 2 | 26/27 | 96.3 | 46.27 (32.13, 60.41) | 40.0 [4-128] | 9 | 1 |
| CM095 | 23.4 | 6 | 43/57 | 75.4 | 3.91 (3.13, 4.68) | 4.0 [1-12] | 12 | 6 |
| **CM096** | 98.5 | 6 | 55/70 | 78.6 | 11.20 (8.96, 13.44) | 9.0 [1-36] | 12 | 3 |
| CM097 | 12.9 | 10 | 15/47 | 31.9 | 1.13 (0.94, 1.33) | 1.0 [1-2] | 12 | 6 |
| CM099 | 48.0 | 5 | 11/24 | 45.8 | 2.45 (1.32, 3.59) | 2.0 [1-6] | 12 | 3 |
| CM102 | 39.2 | 4 | 21/58 | 36.2 | 1.52 (1.13, 1.92) | 1.0 [1-3] | 12 | 4 |
| CM105 | 218.8 | 2 | 39/44 | 88.6 | 62.95 (50.96, 74.94) | 56.0 [2-158] | 12 | 2 |
| CM106 | 256.9 | 2 | 65/67 | 97.0 | 71.13 (56.74, 85.51) | 65.0 [1-209] | 12 | 1 |
| CM108 | 122.2 | 5 | 27/40 | 67.5 | 3.67 (2.31, 5.02) | 3.0 [1-16] | 10 | 5 |
| **CM111** | 73.7 | 1 | 30/36 | 83.3 | 6.53 (4.60, 8.46) | 4.5 [1-18] | 10 | 2 |
| **CM112** | 47.1 | 7 | 35/41 | 85.4 | 10.00 (7.93, 12.07) | 9.0 [1-25] | 12 | 6 |
| CM113 | 40.0 | 1 | 37/48 | 77.1 | 2.78 (2.13, 3.43) | 2.0 [1-9] | 12 | 2 |
| CM115 | 49.0 | 3 | 31/44 | 70.5 | 5.06 (3.56, 6.57) | 4.0 [1-15] | 12 | 4 |
| C029 | 321.37 | 2 | 41/65 | 63,08 | 54.51 (32.93, 76.10) | 27.0 [1-283] | nd | nd |
| C030 | 56.00 | 2 | 20/33 | 60,61 | 5.40 (3.31, 7.49) | 4.0 [1-17] | nd | nd |
| C031 | 43.51 | 3 | 22/44 | 50,00 | 6.27 (3.57, 8.98) | 4.0 [1-25] | nd | nd |
| C032 | 32.52 | 2 | 18/44 | 40,91 | 4.67 (3.11, 6.23) | 3.0 [1-11] | nd | nd |
| C033 | 68.18 | 2 | 28/58 | 48,28 | 5.07 (3.34, 6.81) | 3.0 [1-16] | nd | nd |
| C034 | 207.29 | 3 | 24/44 | 54,55 | 26.25 (14.27, 38.23) | 15.5 [1-106] | nd | nd |
| C035 | 37.04 | 5 | 22/43 | 51,16 | 3.27 (1.43, 5.12) | 2.0 [1-21] | nd | nd |
| C036 | 1107.14 | 3 | 40/51 | 78,43 | 16.75 (12.02, 21.48) | 12.0 [1-66] | nd | nd |
| C037 | 67.67 | 1 | 40/42 | 95,24 | 12.90 (10.26, 15.54) | 11.5 [1-46] | nd | nd |
| C039 | 155.20 | 1 | 48/50 | 96,00 | 20.71 (17.90, 23.52) | 20.0 [1-40] | nd | nd |
| C043 | 223.87 | 1 | 47/52 | 90,38 | 23.15 (20.13, 26.17) | 22.0 [4-43] | nd | nd |
| C044 | 38.46 | 3 | 24/59 | 40,68 | 2.42 (1.60, 3.23) | 2.0 [1-11] | nd | nd |
| C046 | 259.26 | 2 | 36/40 | 90,00 | 18.86 (13.24, 24.48) | 16.5 [1-73] | nd | nd |
| C047 | 123.64 | 2 | 20/31 | 64,52 | 6.25 (4.40, 8.10) | 5.5 [1-16] | nd | nd |
| C048 | 82.54 | 1 | 36/41 | 87,80 | 12.33 (7.56, 17.11) | 7.5 [1-55] | nd | nd |
| C050 | 80.00 | 2 | 49/49 | 100,0 | 31.65 (26.50, 36.81) | 29.0 [2-84] | nd | nd |
| C051 | 51.95 | 3 | 36/46 | 78,26 | 6.17 (4.50, 7.83) | 5.0 [1-23] | nd | nd |
| C053 | 27.78 | 4 | 17/39 | 43,59 | 2.35 (1.62, 3.08) | 2.0 [1-6] | nd | nd |
| C054 | 18.93 | 4 | 21/43 | 48,84 | 1.48 (1.06, 1.90) | 1.0 [1-5] | nd | nd |
| C055 | 214.81 | 4 | 32/39 | 82,05 | 25.25 (17.65, 32.85) | 19.5 [1-82] | nd | nd |
| C056 | 72.73 | 4 | 27/38 | 71,05 | 10.78 (7.99, 13.56) | 10.0 [1-30] | nd | nd |
| C057 | 37.04 | 2 | 39/57 | 68,42 | 2.64 (2.10, 3.18) | 2.0 [1-7] | nd | nd |
| C059 | 59.26 | 3 | 47/52 | 90,38 | 10.53 (8.20, 12.87) | 8.0 [1-44] | nd | nd |
| C060 | 20.00 | 3 | 23/44 | 52,27 | 3.22 (2.44, 4.00) | 3.0 [1-7] | nd | nd |
| C061 | 39.68 | 2 | 36/60 | 60,00 | 3.17 (2.31, 4.03) | 2.0 [1-10] | nd | nd |
| C063 | 118.69 | 4 | 34/49 | 69,39 | 26.09 (21.07, 31.10) | 21.0 [2-61] | nd | nd |
| C064 | 52.76 | 3 | 25/51 | 49,02 | 4.00 (2.48, 5.52) | 3.0 [1-18] | nd | nd |
| C065 | 55.17 | 6 | 31/40 | 77,50 | 5.42 (3.73, 7.11) | 4.0 [1-21] | nd | nd |
| C066 | 70.93 | 2 | 37/45 | 82,22 | 12.68 (9.48, 15.87) | 11.0 [1-46] | nd | nd |
| C067 | 47.13 | 2 | 50/56 | 89,29 | 7.28 (5.87, 8.69) | 6.0 [1-28] | nd | nd |
| C069 | 60.60 | 6 | 42/55 | 76,36 | 5.57 (4.50, 6.64) | 4.5 [1-15] | nd | nd |
| C075 | 170.21 | 1 | 31/33 | 93,94 | 12.58 (9.43, 15.73) | 11.0 [1-36] | nd | nd |
| C077 | 36.33 | 2 | 28/46 | 60,87 | 3.07 (2.39, 3.75) | 3.0 [1-8] | nd | nd |
| C080 | 219.70 | 3 | 66/68 | 97,06 | 33.24 (28.43, 38.06) | 31.5 [1-113] | nd | nd |
| C089 | 24.24 | 2 | 39/51 | 76,47 | 4.33 (3.30, 5.37) | 3.0 [1-14] | nd | nd |
| C091 | 33.67 | 2 | 73/78 | 93,59 | 12.22 (10.73, 13.71) | 12.0 [1-31] | nd | nd |
| C092 | 15.36 | 2 | 43/60 | 71,67 | 5.16 (3.94, 6.38) | 4.0 [1-17] | nd | nd |
| C096 | 25.97 | 2 | 25/55 | 45,45 | 2.68 (1.97, 3.39) | 2.0 [1-7] | nd | nd |
| C097 | 11.83 | 2 | 33/47 | 70,21 | 2.73 (2.18, 3.28) | 3.0 [1-6] | nd | nd |
| C098 | 239.52 | 2 | 59/60 | 98,33 | 60.69 (51.86, 69.53) | 56.0 [3-136] | nd | nd |
| C099 | 71.33 | 2 | 41/61 | 67,21 | 13.39 (10.07, 16.71) | 11.0 [1-56] | nd | nd |
| C100 | 23.19 | 5 | 22/70 | 31,43 | 1.55 (1.21, 1.88) | 1.0 [1-3] | nd | nd |
| C101 | 54.42 | 3 | 107/123 | 86,99 | 15.81 (13.60, 18.03) | 14.0 [1-56] | nd | nd |
| CM003 | 87.50 | 2 | 19/45 | 42,22 | 8.89 (5.05, 12.74) | 6.0 [1-29] | nd | nd |
| CM008 | 16.67 | 3 | 31/60 | 51,67 | 8.19 (5.86, 10.53) | 7.0 [1-26] | nd | nd |
| CM010 | 177.38 | 2 | 19/29 | 65,52 | 26.74 (16.68, 36.80) | 21.0 [2-76] | nd | nd |
| CM011 | 32.65 | 2 | 20/50 | 40,00 | 8.45 (5.63, 11.27) | 7.5 [1-21] | nd | nd |
| CM016 | 91.35 | 2 | 8/19 | 42,11 | 5.00 (2.54, 7.46) | 5.5 [1-9] | nd | nd |
| CM018 | 146.15 | 1 | 18/32 | 56,25 | 49.94 (36.03, 63.86) | 55.0 [1-89] | nd | nd |
| CM019 | 95.24 | 3 | 28/31 | 90,32 | 82.89 (64.34, 101.45) | 84.0 [1-175] | nd | nd |
| CM020 | 82.76 | 3 | 32/49 | 65,31 | 70.28 (54.86, 85.71) | 64.5 [3-168] | nd | nd |
| CM021 | 20.20 | 3 | 48/79 | 60,76 | 3.85 (3.16, 4.55) | 3.0 [1-11] | nd | nd |
| CM022 | 22.86 | 4 | 22/36 | 61,11 | 5.09 (3.57, 6.62) | 4.5 [1-16] | nd | nd |
| CM023 | 15.69 | 2 | 28/39 | 71,79 | 4.29 (3.06, 5.51) | 3.0 [1-12] | nd | nd |
| CM028 | 20.69 | 3 | 14/25 | 56.00 | 9.79 (4.96, 14.61) | 6.0 [1-32] | nd | nd |
| CM029 | 11.90 | 3 | 39/60 | 65.00 | 8.03 (6.51, 9.55) | 8.0 [1-18] | nd | nd |
| CM030 | 96.30 | 2 | 13/23 | 56,52 | 27.00 (12.41, 41.59) | 21.0 [1-95] | nd | nd |
| CM033 | 39.22 | 3 | 16/32 | 50.00 | 6.19 (4.15, 8.22) | 6.0 [1-11] | nd | nd |
| CM034 | 92.86 | 3 | 12/32 | 37,50 | 4.00 (1.70, 6.30) | 2.0 [1-14] | nd | nd |
| CM036 | 17.78 | 3 | 41/45 | 91,11 | 6.41 (5.06, 7.77) | 5.0 [1-16] | nd | nd |
| CM038 | 166.67 | 3 | 39/58 | 67,24 | 6.64 (4.27, 9.01) | 4.0 [1-31] | nd | nd |
| CM039 | 23.08 | 3 | 24/66 | 36,36 | 3.58 (2.30, 4.86) | 3.0 [1-15] | nd | nd |
| CM040 | 213.33 | 4 | 47/63 | 74,60 | 17.21 (12.70, 21.73) | 13.0 [1-56] | nd | nd |
| CM041 | 144.44 | 5 | 26/46 | 56,52 | 9.31 (6.24, 12.38) | 7.0 [1-32] | nd | nd |
| CM044 | 130.72 | 2 | 10/22 | 45,45 | 5.10 (2.58, 7.62) | 3.5 [1-13] | nd | nd |
| CM051 | 140.35 | 6 | 23/30 | 76,67 | 23.13 (13.83, 32.43) | 14.0 [1-89] | nd | nd |
| CM055 | 72.00 | 1 | 52/64 | 81,25 | 15.69 (13.20, 18.18) | 15.0 [1-44] | nd | nd |
| CM058 | 63.49 | 4 | 28/46 | 60,87 | 12.04 (7.12, 16.96) | 6.5 [1-53] | nd | nd |
| CM065 | 407.84 | 1 | 55/64 | 85,94 | 27.65 (21.28, 34.03) | 23.0 [1-121] | nd | nd |
| CM073 | 51.85 | 3 | 48/64 | 75,00 | 7.83 (6.21, 9.45) | 6.0 [1-27] | nd | nd |
| CM074 | 38.46 | 2 | 44/63 | 69,84 | 4.16 (3.29, 5.02) | 3.0 [1-12] | nd | nd |
| CM084 | 50.79 | 5 | 55/59 | 93,22 | 9.44 (8.01, 10.87) | 9.0 [1-23] | nd | nd |
| CM091 | 44.44 | 5 | 41/64 | 64,06 | 2.83 (2.27, 3.39) | 2.0 [1-8] | nd | nd |
| CM103 | 31.25 | 7 | 13/43 | 30,23 | 2.00 (1.46, 2.54) | 2.0 [1-4] | nd | nd |
| CM107 | 22.22 | 3 | 14/30 | 46,67 | 2.43 (1.64, 3.22) | 2.0 [1-5] | nd | nd |
| CM114 | 35.71 | 1 | 26/47 | 55,32 | 2.31 (1.90, 2.71) | 2.0 [1-4] | nd | nd |

**dpi,** days post-infection; **MOI**, multiplicity of infection (minimum number of clones within the parasite isolate); **n/N**, number of infected mosquitoes/number of dissected mosquitoes; **IP**, prevalence of infection (percentage of mosquitoes with at least one oocyst developed in the midgut at day 8 post-infection); **II**, infection intensity (mean number of oocysts per gut) and 95%CI; **median**, median of oocsyts per midgut and range; **N**, number of dissected salivary glands. Gametocyte density is given as the number of sexual parasites per µl of blood.
